# Rapid Extrusion of Myotube-Scale Alginate Fibers for Structured Cultivated Meat

**DOI:** 10.1038/s41538-026-00994-2

**Published:** 2026-07-21

**Authors:** Alex S. Kermani, Andrea C. Filler, Edrin C. Miller, David E. Block, J. Kent Leach

**Affiliations:** 1 Department of Orthopaedic Surgery, UC Davis Health, Sacramento, CA 95817 USA; 2 Department of Viticulture and Enology, UC Davis, Davis, CA 95616 USA; 3 Department of Chemical Engineering, UC Davis, Davis, CA 95616 USA; 4 Department of Materials Science and Engineering, UC Davis, Davis, CA 95616 USA; 5 Department of Biomedical Engineering, UC Davis, Davis, CA 95616 USA

**Keywords:** Biological techniques, Biotechnology, Engineering, Materials science

## Abstract

Cultivated meat (CM) scaffolds must be edible and produced with high throughput. This study introduces a novel approach for structured CM production using alginate-based scaffolds prepared using a liquid-liquid extrusion method called Pressurized Apparatus for Striated Tissue Assembly (PASTA). We hypothesized that PASTA scaffolds could enable the creation of tissue constructs exceeding 500 µm thickness with food-scale throughput. Leveraging sub-boiling hydrothermal degradation, we modulated the biophysical properties of alginate, resulting in controlled reductions in viscosity, zeta potential, and hydrogel mechanical properties. PASTA extrusion consistently produced hydrogel fibers 10–42% smaller than the extrusion outlet diameter. Murine C2C12 myoblasts cultured on PASTA scaffolds modified with adhesive peptides adhered, proliferated, and differentiated along the noodle axis. This was confirmed by a 3.5-fold increase in DNA quantity over 4 days, substantial cell viability after 5 days in culture, late-stage myogenic gene expression (*Myog, Myh2*), and positive immunohistochemistry for myosin heavy chain. PASTA enables rapid, parallelizable production of aligned fiber scaffolds with physiologically relevant dimensions using inexpensive, food-grade alginate. These findings demonstrate a proof-of-concept scalable and modular platform to produce cell-laden tissues for manufacturing structured CM.

## Introduction

Global meat consumption is hundreds of billions of kilograms annually and rising. In 2022, humans across the globe consumed 363 billion kilograms of beef, veal, pork, sheep, and poultry^1,2^. Skeletal muscle represents the vast majority of animal meat by mass and nutritional value^3^. Consuming meat can be beneficial because it provides all nine essential amino acids, key micronutrients like iodine, iron, zinc, and complex B vitamins like B12^4^. However, animal meat production faces uncertainty from seasonal variation^5^, greenhouse gas emissions^6^, pathogenic risk^7^, and supply strained by growing demand^8^.

Cultivated meat (CM) refers to animal meat produced ex vivo^9^. In contrast to ground meat that lacks structure and organization, structured CM is any CM that mimics native tissue structure, like a cultivated ribeye steak. Such products, considered the goal of CM, remain elusive. Moreover, for CM to compete with conventional meat, the field must achieve megatonne (1e9 kg) production and low cost^10^. Using tissue engineering principles to produce CM may offer additional benefits for our food supply by reducing arable land used to produce meat, adding valuable nutrients like dietary fiber, and stabilizing national food supplies from external disruption.

Tissue engineered skeletal muscle typically relies on aligned scaffolds, external stimulation, myogenic cells, or a combination of these factors. Researchers can precisely direct cells and/or materials to create structure^11,12^, but often at the expense of throughput, fiber continuity, and gentle cell handling. Especially for biomedical applications, production throughput is rarely a consideration unless there is a practical limitation (e.g., cell death or evaporation). Published work that investigates fibers often does so with electrospinning^13^ (<0.1 mL per hour), wet spinning^14^ (single mL per hour), or rotary jet spinning^15^ (tens of mL per hour). Typically, researchers rely on the prospect of massive parallelization to achieve substantive quantities of scaffold.

Biomaterials can supplement product mass, provide essential instructive cues to guide cell fate, and add nutritional value to the final product. Natural materials including cotton, silk^16^, zein^17^, pea protein^18^, and many others can be transformed into continuous fibers. Food-grade sodium alginate is an inexpensive polysaccharide derived from brown seaweed, composed of mannuronic acid (M) and guluronic acid (G). These extracts have a high molecular weight, yielding high viscosity alginate solutions. Common methods for reducing alginate molecular weight are irradiation, hydrothermal treatment, acid, base, or oxidative reactions, or enzymatic degradation^19^. Hydrothermal treatment has been used to tune viscoelastic properties of alginate^20^, but this approach has not been widely adopted as a method for viscosity reduction and increased throughput. Hydrothermal treatment is advantageous because it does not require expensive enzymes, harsh chemical conditions, radiation sources, or other specialized equipment, making it attractive, accessible, and realistic as a step in CM production.

**Fig. 1:**
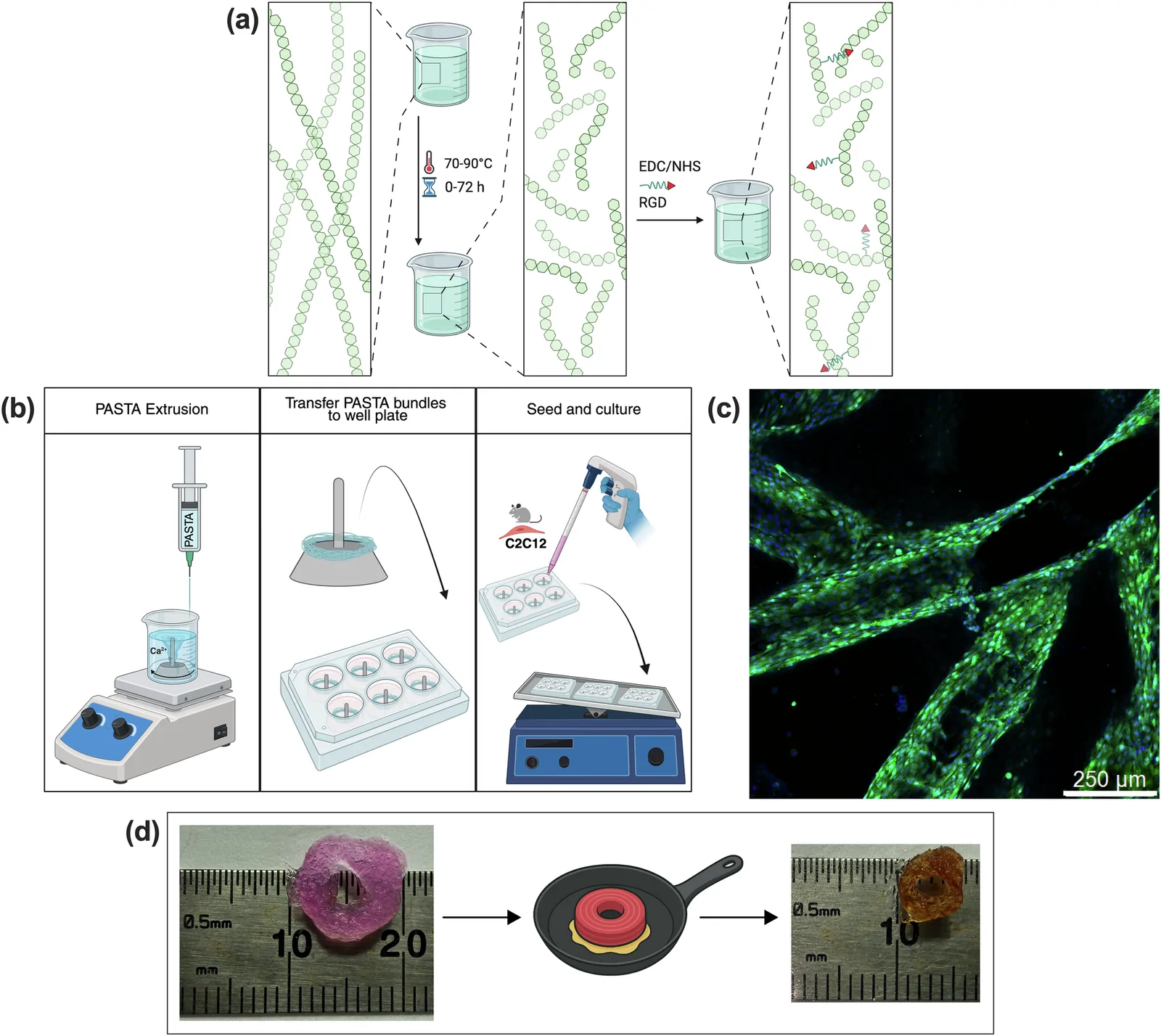
Graphical summary of end-to-end PASTA cultivated meat production. **a** Alginate was first hydrothermally degraded, then RGD-functionalized using established peptide coupling chemistry. The result was degraded, low viscosity, RGD-modified sodium alginate. **b** Alginate was extruded using the PASTA, transferred to well plates, seeded with C2C12 murine myoblasts and cultured under myogenic conditions. **c** C2C12s colonize and align along the PASTA noodle axis. Maximum projection confocal image of live (green) with nuclear counterstained (blue) C2C12s after 5 days in growth medium. **d** One PASTA noodle bundle after 10 days of differentiation culture was pan-fried in vegetable oil until browned.

This work addresses two critical barriers preventing high-volume production of thick, structured CM. First, we propose a rapid alginate extrusion method, pressurized apparatus for striated tissue assembly (PASTA), to produce alginate “noodles.” Second, we collect the PASTA noodles in an aligned bundle, creating scaffold structure that guides myoblast alignment in a pseudo-3D (2.5D) culture system (Fig. 1). PASTA advances the field by forming long, continuous, cell-compatible alginate fibers with tunable mechanical properties. We hypothesized that hydrothermally degraded, food-grade alginate extruded with PASTA would generate continuous, aligned fiber scaffolds with physiologically relevant fiber diameters and food-relevant throughput that support myoblast adhesion, proliferation, and myogenic differentiation for structured cultivated meat production. The results of this study represent proof of concept that PASTA bundle culture can enable high-throughput, structured CM.

## Results

### Hydrothermal degradation effectively alters biophysical properties of alginate

**Fig. 2:**
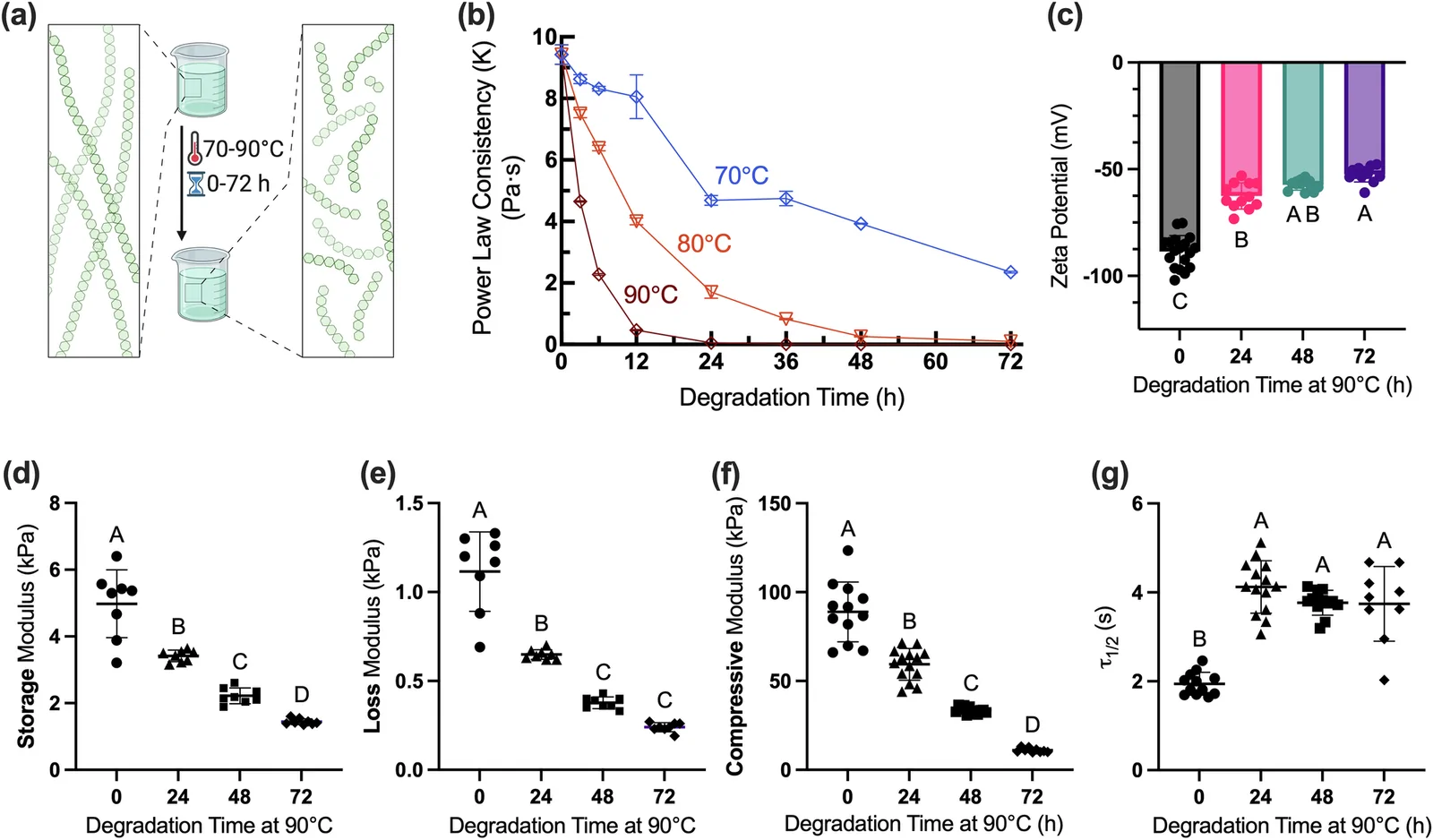
Hydrothermal degradation time modulates the biophysical properties of alginate and derived hydrogels. **a** Alginate solutions (1% w/v) were heated at 70 °C, 80 °C, or 90 °C for up to 72 h, **b** resulting in a temperature- and time-dependent decrease in viscosity, with the most rapid reduction observed at 90 °C (*n* = 3). **c** Zeta potential magnitude of 0.2% w/v solutions decreased with degradation time at 90 °C (*n* = 12–18). Hydrogels formed from degraded alginate exhibited reductions in both **d** storage modulus and **e** loss modulus after 72 h degradation (*n* = 8). **f** Similarly, compressive modulus decreased with increasing degradation time (*n* = 9–14). **g** Stress relaxation half-time (τ_1/2_) at 15% strain increased following degradation (*n* = 9–14) but did not differ substantially after 24 h. Different letters denote significant differences between groups.

To extrude alginate solutions at a reasonable pressure, degradation was evaluated to reduce solution viscosity (Fig. 2a). Aqueous alginate solutions became less viscous when heated at temperatures between 70 and 90 °C for 3 days (Fig. 2b). The consistency (K) of an undegraded 1% w/v solution of alginate was 9.4 ± 0.3 Pa s^*n*^ (*n* = 3). The time to reach half the original viscosity for samples at 70 °C, 80 °C, and 90 °C was greater than 28 h, 10 h, and 3 h, respectively. At 24 h of degradation, the consistency of the samples at 70 °C, 80 °C, and 90 °C was 4.7 ± 0.2 Pa s^*n*^, 1.7 ± 0.2 Pa s^*n*^, and 0.05 ± 7e−4 Pa s^*n*^ (*n* = 3 for each group), respectively. The complete data set is shown in Supplementary Data 1. Subsequent analysis was focused on alginate thermally degraded at 90 °C for 24 h.

We performed gel permeation chromatography (GPC) to quantify the reduction in molecular weight before and after our target degradation treatment (90 °C, 24 h). The number average molecular weight (*M*_N_) decreased by 61%, weight average molecular weight (*M*_W_) decreased by 75%, the z average molecular weight (*M*_Z_) decreased by 85%, the molecular weight at peak maximum (or mode molecular weight), (*M*_P_) decreased by 61%, and the molecular mass dispersity (*Đ*_M_) decreased by 34%. See Table 1 below.

**Table 1:** GPC results for 1% w/v alginate solutions

Sample	*M*_N_ (Da)	*M*_W_ (Da)	*M*_Z_ (Da)	*M*_P_ (Da)	*Đ*_M_
Undegraded	257,266	994,158	3,669,822	420,415	3.87
Degraded at 90 °C for 24 h	99,354	251,804	553,933	163,124	2.54

Proton NMR (Supplementary Data 2) of the undegraded alginate revealed the fraction of G residues (*F*_G_) was 0.43 and the fraction of M residues (*F*_M_) was 0.57. After 24 h of hydrothermal degradation at 90 °C and RGD modification, the fraction of G residues (*F*_G_) was 0.51 and the fraction of M residues (*F*_M_) was 0.49. Supplementary Data 3 lists the complete quantification of M and G fractions.

Alginate solution zeta potential decreased over the course of degradation at 90 °C (Fig. 2c). The zeta potential of an undegraded 0.2% w/v alginate solution was −89 ± 7.6 mV (*n* = 18). After 24, 48, and 72 h of degradation, the zeta potential decreased in magnitude to −63 ± 6.1 mV (*n* = 12), −57 ± 2.5 mV (*n* = 12), then −52 ± 3.6 mV (*n* = 12), respectively. These data suggest the polymer chain length decreases during hydrothermal degradation.

The storage and loss modulus of gels made with degraded alginate decreased with increasing degradation time (Fig. 2d, e). The average storage and loss modulus of undegraded alginate gels was 5.0 ± 1.0 kPa and 1.1 ± 0.20 kPa, respectively. After 24 h of hydrothermal degradation at 90 °C, the storage modulus was 3.4 ± 0.17 kPa and the loss modulus was 0.65 ± 0.03 kPa. The storage modulus continued to decrease, reaching 1.4 ± 0.09 kPa after 72 h of degradation. The loss modulus after 72 h of degradation was 0.24 ± 0.03 kPa and was not statistically different from the loss modulus at 48 h (0.38 ± 0.03 kPa, *n* = 8 for all groups) according to the one-way ANOVA analysis. Our data suggest that degradation time is inversely related to storage and loss modulus.

The compressive modulus of degraded alginate gels is shown in Fig. 2f. The compressive modulus of undegraded alginate gels was 89 ± 17 kPa (*n* = 12). After 24, 48, and 72 h of degradation, the compressive modulus was 59 ± 8.9 kPa (*n* = 14), 33 ± 2.0 kPa (*n* = 12), and 11 ± 1.2 kPa (*n* = 9), respectively. Similarly to the storage and loss modulus data, this demonstrates that degradation time is inversely related to the bulk compressive modulus.

To measure the stress relaxation behavior of the degraded alginate gels, we reported the time it takes (in seconds) to dissipate half of the applied stress (τ_1/2_, Fig. 2g). The undegraded alginate gels had a τ_1/2_ of 1.9 ± 0.26 s (*n* = 12). The 24, 48, and 72 h degraded alginate gels had a τ_1/2_ of 4.1 ± 0.59 s (*n* = 14), 3.8 ± 0.28 s (*n* = 12), and 3.7 ± 0.84 s (*n* = 9), respectively.

Collectively, our data demonstrate the reduction of polymer chain length during hydrothermal degradation; the inverse relationship between degradation time and storage, loss, and compressive modulus; and rapid stress relaxation.

### PASTA throughput and noodle diameter is impacted by extrusion and stir speed

**Fig. 3:**
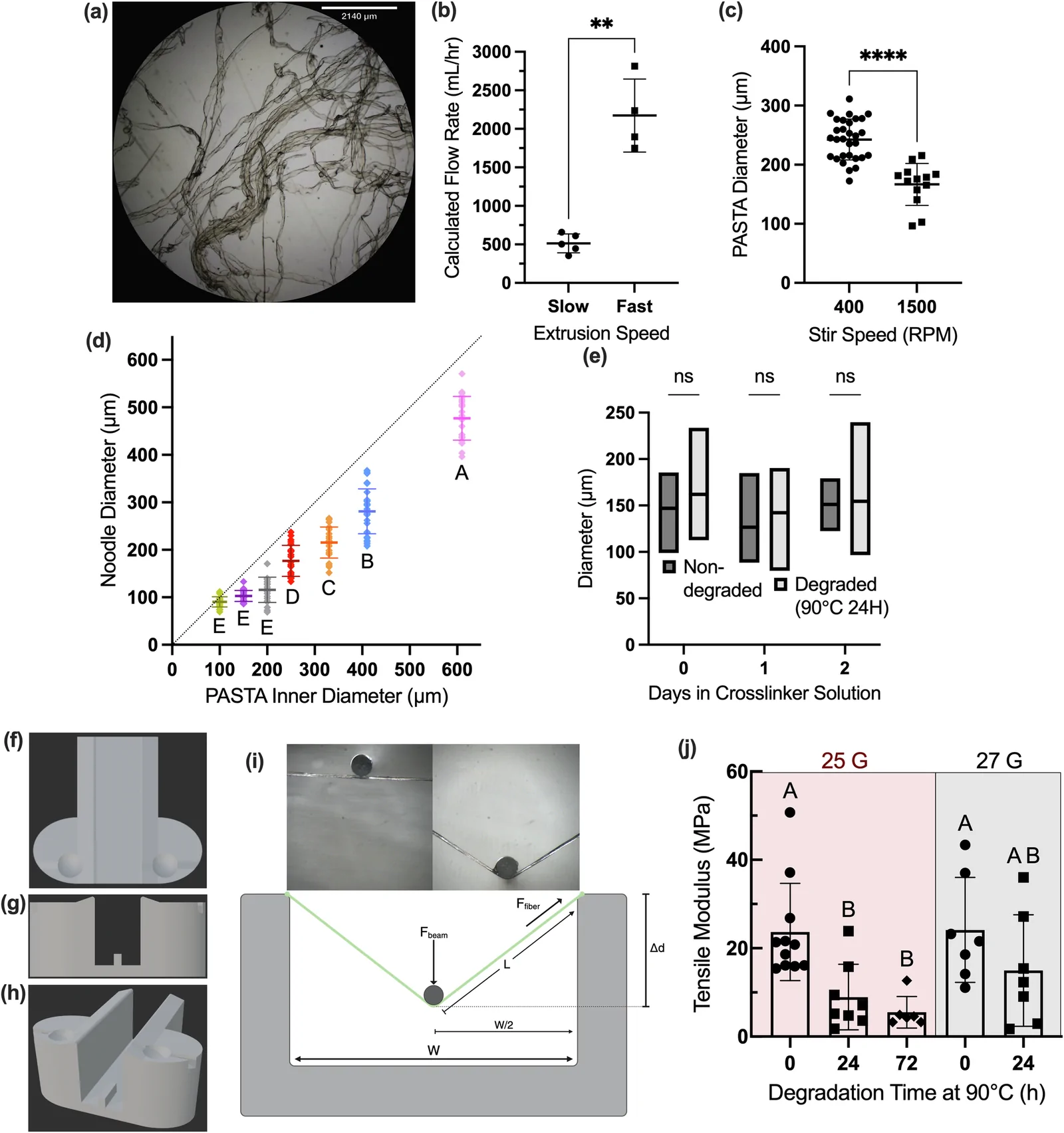
Diameter and mechanical properties of PASTA noodles are governed by extrusion conditions and polymer degradation. **a** Representative brightfield image of PASTA noodles extruded with a 27 G outlet (200 µm I.D.). **b** Hourly flow rate of PASTA extruder at slow and fast manual extrusion speeds using a 27 G outlet and degraded alginate solution (90 °C, 24 h). Two-tailed *t*-test *p* < 0.005. **c** PASTA diameter as a function of collector stir speed using a 25 G outlet. Two-tailed *t*-test *p* < 0.0001. **d** PASTA noodle diameters as a function of inner diameter (*n* = 24). Dotted line is *y* = *x*. See Supplementary Data 4 for detailed size measurements. **e** Diameter of PASTA fabricated from undegraded or hydrothermally degraded (90 °C, 24 h) alginate using a 27 G outlet (*n* = 24). Mean with min and max, unpaired *t*-test with Welch correction. **f** Top (**g**) Front and **h** Angled view of tensile testing jig. **i** Images and free body diagram of tensile testing method. A single noodle is suspended across a gap and strained. **j** Mean tensile modulus of PASTA noodles (25 G, 250 µm I.D. and 27 G, 200 µm I.D.) (*n* = 6–11). Different letters denote significant differences between groups.

Extruded PASTA noodles are long, continuous fibers (Fig. 3a) that can range in diameter and roughness. To quantify the theoretical PASTA throughput, we measured the volumetric flow rate at a slow and fast extrusion speed. Slow PASTA extrusion had a mean flow rate of 514 mL/h and fast PASTA extrusion had a mean flow rate of 2173 mL/h (Fig. 3b), with a maximum measured throughput of 2813 mL/h. The collector stir speed was inversely related to the PASTA noodle diameter (Fig. 3c). A low stir speed of 400 RPM yielded a mean diameter of 242 µm and a high stir speed of 1500 RPM yielded a mean diameter of 167 µm from a 25 G (250 µm I.D.) outlet.

### Diameter of PASTA noodles is consistently smaller than the extrusion outlet

We can reliably produce PASTA noodles that have a diameter less than the extrusion outlet diameter. Across a range of outlet diameters between 100 µm and 610 µm, the resulting PASTA noodle diameters were between 10% and 42% smaller than the outlet diameter, with the smallest change in diameter at the smallest outlet. A 200 µm outlet was chosen for PASTA bundle culture experiments and the resulting noodles had a mean diameter of 116 µm (Fig. 3d). Since myoblasts are tens of microns in diameter and myotubes are hundreds of microns in length, we expect this noodle diameter to effectively constrain the cell orientation.

PASTA was prepared from undegraded and degraded (90 °C, 24 h) alginate with a 27 G (200 µm I.D.) needle tip. For undegraded alginate noodles, the average noodle diameter was between 124 and 151 µm. In the case of degraded alginate, the average noodle diameter was between 142 and 162 µm (Fig. 3e). The diameter of both groups of PASTA noodles did not change over the course of 2 days in crosslinker, nor did the degraded and undegraded groups significantly differ in mean diameter.

### Mechanical properties of PASTA noodles are influenced by hydrothermal degradation

The tensile modulus of PASTA noodles decreased with increasing degradation time (Fig. 3j). Using the 25 G outlet, the tensile modulus of the 1% w/v alginate noodles was 23.7 ± 11.0 MPa (*n* = 11). After 24 h of degradation at 90 °C, the tensile modulus was 8.9 ± 7.4 MPa (*n* = 8). After 72 h of degradation, the tensile modulus was 5.5 ± 3.6 MPa (*n* = 6). Using a 27 G outlet, the undegraded tensile modulus was 24 ± 12 MPa (*n* = 7) and 15 ± 13 MPa (*n* = 7) after degradation at 90 °C for 24 h. Noodles did not form at 72 h of degradation using the 27 G outlet. These data demonstrate that PASTA noodle tensile modulus can be tuned with degradation time, and most of the tensile modulus reduction happens in the first 24 h of degradation at 90 °C.

### C2C12s adhere to, proliferate on, and colonize PASTA noodle scaffolds

**Fig. 4:**
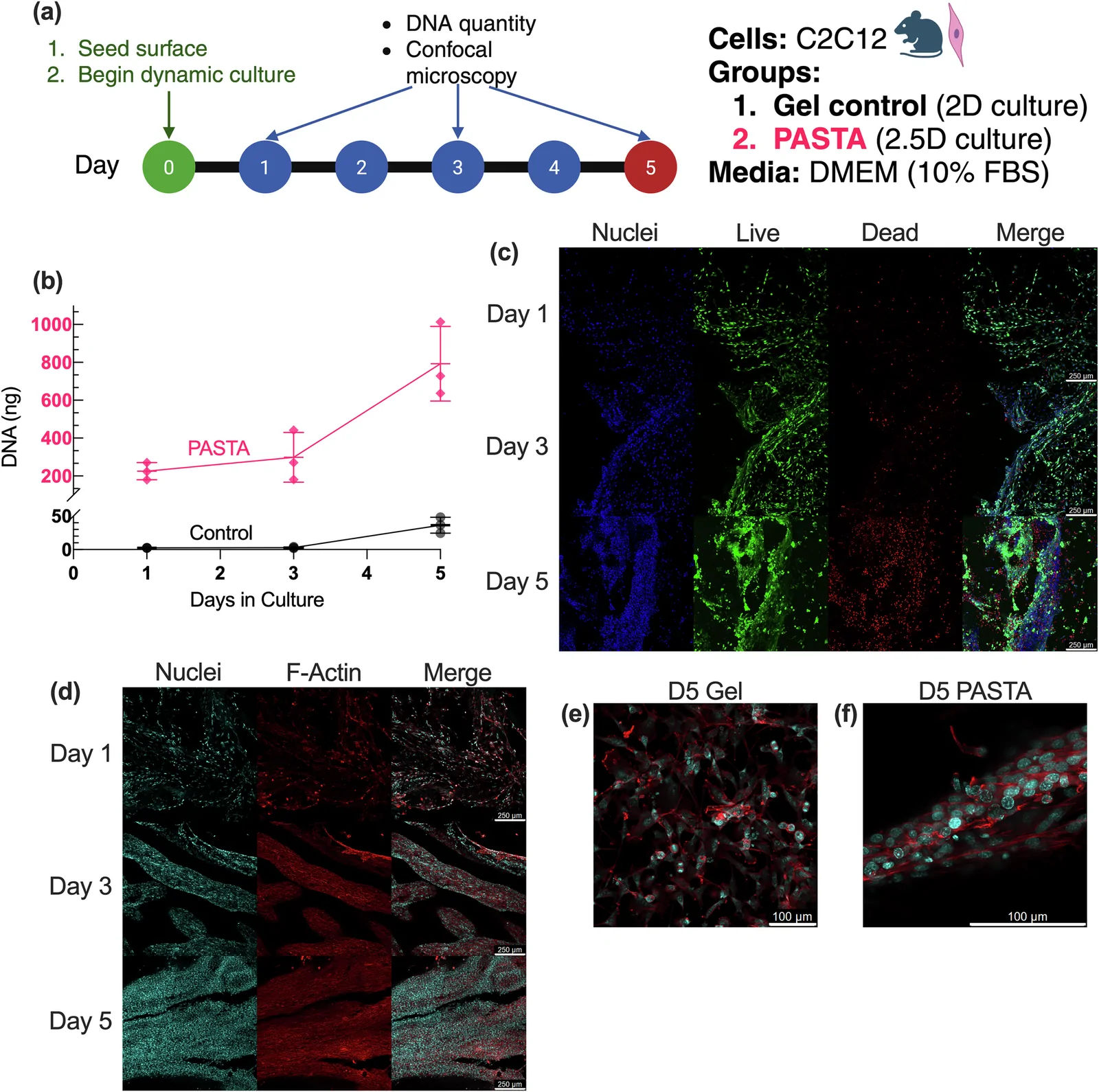
C2C12s attach and proliferate on PASTA scaffolds. **a** Experiment schematic. C2C12s were cultured in growth medium for 5 days on PASTA bundles and alginate gels (Control). DNA quantity and confocal images were taken at day 1, day 3, and day 5. **b** DNA quantity increased 3.5-fold on PASTA over 4 days compared to 15-fold for the control. **c** Live/dead stain of PASTA scaffolds throughout culture. Cells are alive and colonizing PASTA noodles, though dead cells are also increasing by D5. **d** Nucleus (DAPI) and F-actin (phalloidin) stain of PASTA scaffolds. Scale bar is 250 µm. **e** C2C12s are randomly oriented when cultured on Gels and **f** aligned along the noodle axis when cultured on PASTA. Scale bar is 100 µm.

To assess the suitability of this system for muscle cell culture, we investigated the proliferation of C2C12 murine myoblasts on PASTA scaffolds (2.5D culture) and alginate gel discs (2D culture). The experiment schematic is shown in Fig. 4a.

C2C12s adhered and proliferated on the PASTA noodle scaffolds due to interaction with the covalently linked RGD adhesion peptide. We measured changes in DNA content over time as an indicator of cell proliferation (Fig. 4b). DNA content of C2C12s seeded on alginate gels (Control) increased from 2.45 ± 0.104 ng (*n* = 3) at D1 to 36.5 ± 11.8 ng (*n* = 3) after 5 days in static culture, a doubling time of 24 h. The DNA content of PASTA bundles increased from 225 ± 45.3 ng (*n* = 3) at D1 to 793 ± 197 ng (*n* = 3) after 5 days in dynamic culture, a doubling time of 53 h.

Cell spreading and colonization of PASTA scaffolds was assessed using live/dead stain (Fig. 4c) and nucleus/F-actin stain (Fig. 4d). Confocal imaging of live/dead samples showed increasing density of both live and dead cells. The cells appeared to attach and align along the noodle axis. Nucleus and F-actin stain show the same increased cell density and alignment (Fig. 4d, f) compared to the cells on a 2D surface (Fig. 4e).

### C2C12s differentiate on PASTA noodle scaffolds

**Fig. 5:**
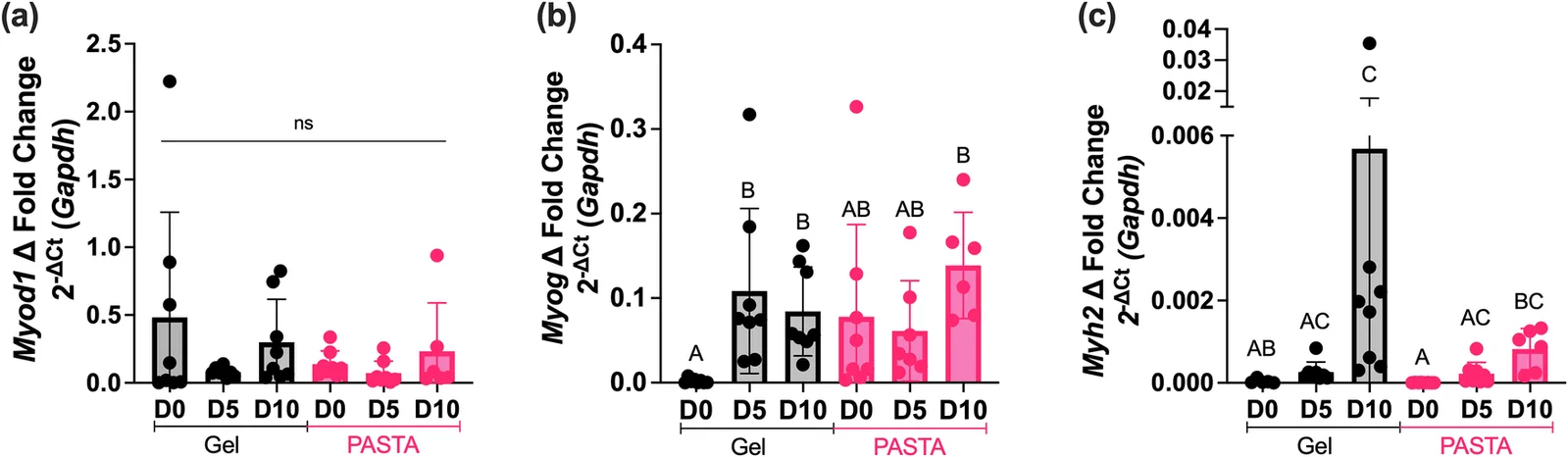
RT-qPCR suggests late-stage myogenic gene expression is increasing for both groups during culture and has earlier onset with PASTA. **a**–**c** PCR single delta fold change (2^−∆Ct^) with respect to *Gapdh* with murine *Myod1*, *Myog*, and *Myh2* for both Gels and PASTA experiment groups. Compact letter display shows mean rank differences of ∆Ct (not fold change) calculated using the Kruskal-Wallis test with Dunn’s multiple comparison tests and alpha = 0.05.

With an eye toward inducing muscle tissue formation on PASTA noodle scaffolds, we investigated the capacity of C2C12s to undergo differentiation by assessing gene expression and protein deposition on an alginate gel surface compared to PASTA scaffolds. Gene expression of murine *Myod1*, *Myog*, and *Myh2* was quantified at days 0, 5, and 10 of differentiation with real-time quantitative polymerase chain reaction (RT-qPCR) (Fig. 5a–c). For *Myod1*, there were no significant differences between the experimental groups or time points. For the Gel group, *Myog* increased between D0 and D5, then maintained through D10. Myoblasts on PASTA, conversely, exhibited higher *Myog* expression initially and maintained that level throughout the experiment. *Myh2* expression progressively increased in both Gel and PASTA groups. While not statistically significant, the average expression in Gels is higher than in PASTA by D10.

**Fig. 6:**
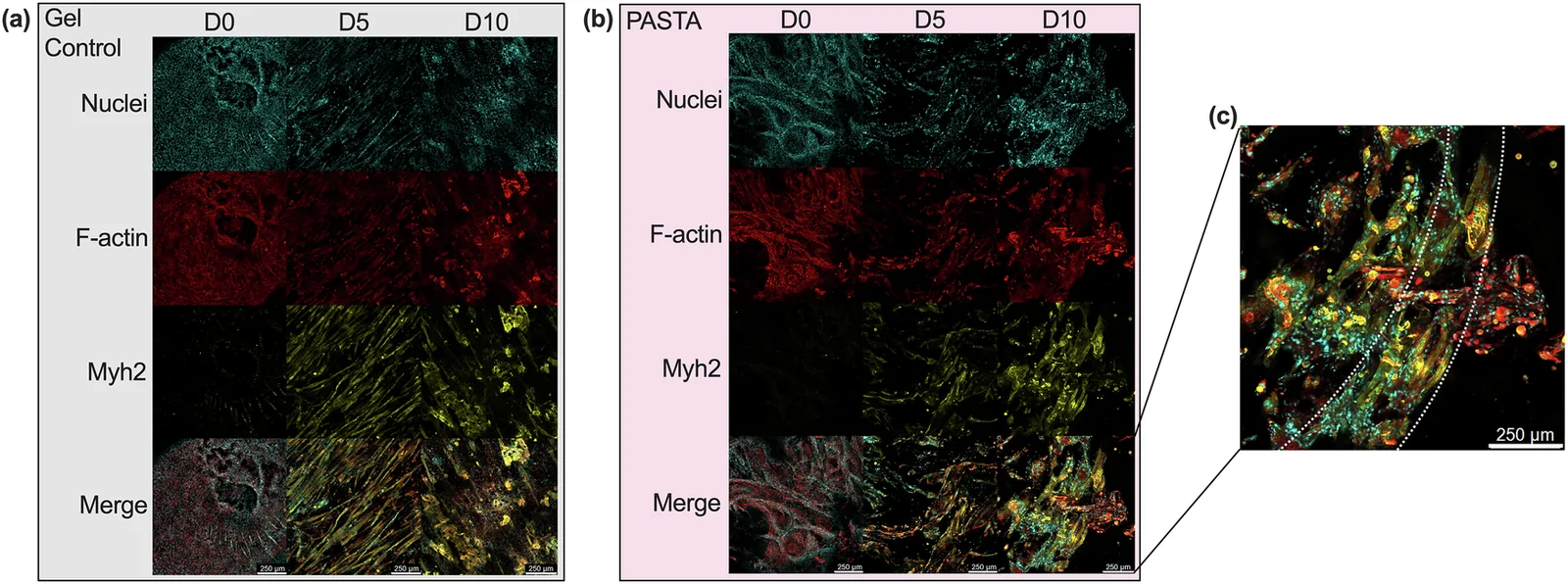
C2C12s on PASTA scaffolds exhibit markers of late-stage myogenic protein deposition. **a**, **b** In both groups, minimal protein signal was observed at D0; by D5, clear myotube formation and alignment was detected. At D10, Gel control myotubes largely persisted but showed partial detachment, while PASTA-cultured myotubes increased in size and maintained alignment along the noodle axis. Representative images shown for each time point. **c** Magnified image of PASTA D10 Merge with a white outline of an individual PASTA noodle. Scale bar is 250 µm.

We used immunohistochemistry for myosin heavy chain to reveal the progressive development of the late-stage myogenic protein during 10 days of culture in differentiation media for both Gel control (2D culture) and PASTA (2.5D culture) groups (Fig. 6). Minimal signal was present at D0 for cells on both substrates. By D5, there was clear protein expression and alignment of myotubes, especially in the Gel group. By D10, the myotubes appeared to have partially detached from the culture surface, but the intensity and area of the stain were largely maintained. Especially in the PASTA group, myotubes appeared to increase in size relative to D5.

## Discussion

This study addressed a critical limitation in CM production: the poor throughput of current CM scaffold production methods. We hypothesized that PASTA noodle scaffolds could enable structured CM by rapidly creating aligned tissue culture scaffolds with total dimensions exceeding typical diffusion limits (i.e., 500 µm). We investigated the feasibility of PASTA scaffolds for structured CM by first exploring a sub-boiling hydrothermal degradation process to tune the properties of alginate solutions and their resulting hydrogels. We then utilized a high-throughput liquid-liquid extrusion apparatus (PASTA) to produce fibers (“noodles”) as small as 40 µm using hydrothermally degraded alginate. Our results validated this approach: sub-boiling hydrothermal degradation effectively influences alginate viscosity, zeta potential, and hydrogel mechanical properties. PASTA consistently produced hydrogel noodles with diameters smaller than the extrusion outlet, and C2C12s readily proliferated, colonized, and differentiated on the resulting aligned scaffolds.

Commercial alginate extracts are typically high molecular weight (Mw > 100 kDa) and form high viscosity solutions, though polymer weight range depends on the specific extraction method^21^. Our food grade alginate came from the manufacturer with a number average molecular weight of over 257 kDa. Hydrothermal degradation reduces the polymer chain length by water-mediated scission of the glycosidic linkage^22^, thereby reducing solution viscosity at fixed concentration. Previous literature has reported degradation of sodium alginate at temperatures exceeding atmospheric boiling point^22^ using autoclave cycles^20^, enzymes^23^, ozonation^24^, oxidation^25^, and ultrasonic/ultraviolet/gamma irradiation^26^. Our work expands this range using atmospheric pressure and sub-boiling temperatures, ensuring this process is simple to implement at any scale without additional specialized equipment or reagents. This approach democratizes the capacity to tune alginate biophysical properties. Prior research exploring hydrothermal degradation at 180–240 °C suggests that MM blocks likely decrease relative to GG and MG blocks^22^. GG enrichment would enhance mechanical strength, since the strongest crosslinking interactions happen between opposing GG blocks and calcium ions. Indeed, our ^1^H NMR results (Supplementary Data 3) agree with this literature. For incompressible fluids in laminar flow, the Hagen-Poiseuille equation predicts that lower viscosity solutions require less extrusion pressure (∆*P* ∝ 1/*r*^4^) and enables higher flow rates (∆*P* ∝ Q). Though alginate solutions are known to be non-Newtonian at high shear rates^27^, this principle still guides throughput. In fact, shear thinning further reduces apparent viscosity, increasing volumetric flow in laminar regimes. Extrusion shear can transiently align the polymer chains and increase exposure of linear GG blocks. Subsequent gelation permanently aligns these chains and may benefit the mechanical strength of the outer shell of the PASTA noodle. While low viscosity alginate is beneficial for manufacturing throughput, it must be balanced with gel mechanical properties because excessive degradation yields compliant, brittle gels or prevents gelation entirely.

Previous research confirms the direct relationship between alginate chain length and gel stiffness^28^. The storage modulus of degraded alginate gels (Fig. 2d, 90 °C, 24 h) was 3.4 kPa. Using an estimate for Young’s modulus based on storage modulus, we would expect an elastic modulus of about 10 kPa. Instead, we measured 59 kPa. This discrepancy may relate to the oscillation strain rate (10 rad/s, ~9.5 RPM). Too low an oscillation strain rate allows rapid stress relaxation and artificially low measured response. The extent of degradation can also be used to adjust textural experience if desired, though we do not explore the sensory differences from changes in noodle mechanical properties.

At neutral pH, alginate’s carboxyl groups make it anionic (the pKa of an alkyl carboxyl group is approximately 4), so longer chains have a greater total negative charge. Our zeta potential results demonstrate a reduction in negative charge with increasing degradation, which aligns with previously published literature^20^. Degradation also produces oligomeric alginates (2–25 monomers), with immunomodulatory, antitumor, antioxidant, antihypertensive, and antidiabetic effects^26^, plus soluble organic acids like lactic and glycolic acid^22^. For PASTA, gel formation speed and robustness is critical. Therefore, we selected treatment at 90 °C for 24 h to get the greatest viscosity reduction in the least time, while still maintaining sufficient mechanical strength.

Stress-relaxing biomaterials benefit adherent cell culture compared to highly elastic materials^29,30^. Therefore, our fast relaxation time (~2–4 s) is likely advantageous. However, we were surprised that relaxation time increased from 2 s (no degradation) to 4 s (24-h degradation), without further change upon additional degradation. We expected faster-relaxing gels with increased degradation due to less chain entanglement. Our observation may be explained by a selective reduction of MM blocks (*F*_MM_ = 0.35 for undegraded alginate and *F*_MM_ = 0.26 for degraded and RGD-modified alginate), yielding a network of more elastic GG crosslinking junctions and fewer fast-relaxing pathways. These results merit further investigation, especially to elucidate the relaxation time plateau after 24 h of degradation.

Alginate lacks native cell attachment motifs, so we coupled RGD peptides to hydrothermally degraded alginate. Adhesion peptide density affects cell behavior^31^, so to aid reproducibility, we calculated the number of monomers and targeted a fraction to modify (ignoring the sodium counterion), assuming complete yield. We did not modify our alginate using the degree of substitution (DS) calculation, which relies on the polymer molecular weight. However, for ease of comparison to other literature, we used our GPC data (*M*_N_) to determine that the DS was approximately 10. While we used RGD for cell attachment, Seo et al.^14^ found that using a very high crosslinker concentration (9–11% w/v) yielded alginate hydrogels with “excellent” cell adhesion, suggesting a potential path away from costly, non-food-safe peptide modification.

Fibrous tissue culture scaffold literature shows that tissue-specific hierarchy and organization length scales are valuable guidelines for scaffold design^32^. For skeletal muscle, we chose a “top-down” approach (where structure is deterministic), in part because published work in the 50–500 µm fiber diameter range is scarce. Previous research demonstrates that aligning myoblasts during culture improves myogenesis^33^, so our target diameter for PASTA noodles was about 10-fold greater than the short axis of a myoblast (i.e., 100–200 µm). This diameter presents two vastly different radii of curvature to the cells, which can direct cell orientation, improve alignment, and ultimately benefit myogenesis. This work, however, does not explore cell alignment in detail.

When producing PASTA scaffolds, it was surprising to observe noodle diameters consistently 10–42% smaller than the extrusion outlet diameter. This may be caused by strong ionic interaction between the negatively charged alginate and the positively charged crosslinking ion (Ca^2+^), contracting the gel on formation. In one study, high crosslinker concentrations (>2 mM) caused gel shrinking and low concentrations (1 mM) caused swelling^34^. We use a 500 mM calcium concentration for scaffold production, and 5 mM in growth and differentiation media. Rayleigh instability of a fluid stream also narrows the stream before pinching into droplets. This transition zone can be exploited through adjusting the distance between the PASTA outlet and the crosslinker surface. Early experiments of PASTA extrusion found that the noodle production worked best when the liquid stream traveled through air before contacting the crosslinker bath. If the PASTA outlet was placed inside the crosslinker bath, the needle quickly clogged. PASTA achieves kilograms per hour throughput in a bench-scale configuration. While this is a far cry from a billion kilograms annually, it is 1000× closer than similar technologies and much simpler to implement.

C2C12s attached and proliferated on PASTA scaffolds. Despite a 3.5-fold increase in DNA over four days (Fig. 4b), the cell doubling time in the PASTA group was more than twice that of the Gels. This could result from inhomogeneous seeding or nutrient limitations. Inhomogeneous seeding may cause contact inhibition and a reduction in proliferation rate in the high- density areas. Nutrient limitation may be a factor because at D0 of the proliferation experiment, there were approximately 100,000 cells per mL of medium in the PASTA culture, as opposed to approximately 10,000 cells per mL of medium in Gels. PASTA scaffolds have dimensions exceeding typical diffusion limits, and dynamic culture promotes convective mixing, but cells could still be nutrient limited despite changing culture media every other day.

Live/dead staining corroborated the DNA data, showing increased cell density over 5 days. Qualitatively, both live and dead cell densities increase during PASTA noodle culture. Nucleus and F-actin staining confirmed that C2C12s attach to and align along the noodles in PASTA scaffolds (Fig. 4f), supporting our hypothesis that a noodle diameter between 100 and 200 µm sufficiently cues myoblast alignment. These results correspond with expectations from literature of C2C12 culture on RGD-modified alginate gels. However, there is a dearth of published experimentation with food-grade alginate, which is a key innovation of our work that enables inexpensive polymer libraries for CM instead of highly purified material.

In differentiation experiments, serum-starved medium induced differentiation in both Gel and PASTA groups. IHC staining for Myosin-2 revealed progressively increasing protein expression, consistent with late-stage myogenesis. By D10, both groups exhibited some myotube delamination and clumping, though less so in PASTA. We attribute differentiation to a combination of biochemical and biophysical cues. First, prior work in C57BL/6 mouse skeletal muscle found that substrates with a stiffness similar to healthy muscle tissue (approximately 12 kPa) enhanced myotube formation over 4 weeks^35^. Our rheometric data of degraded alginate PASTA approaches this modulus. Second, it is well known that over-confluent muscle cell cultures can spontaneously differentiate. Our high seeding density was intended to promote cell-cell interactions that trigger differentiation. Third, PASTA scaffolds have two very different radii of curvature, one infinite and one finite. This difference provides a topographical cue for alignment along the noodle axis by restricting cells to a single direction that appears ‘flat’ to myoblasts. RT-qPCR corroborated these observations with modest group differences. In Gels, *Myog* increased from D0 to D5 and then plateaued. In PASTA, *Myog* expression was greater than Gels at D0 but the difference was not statistically significant. *Myh2* expression increased in both groups, with a greater change in Gels but a significant increase in PASTA samples by D10. Overall, transcriptional trends aligned with IHC, though many group or time comparisons were not statistically significant.

While our results are promising, there are notable limitations. For example, despite several attempts to do so, we did not control extrusion pressure or flow rate during PASTA extrusion, relying instead on operator judgment for outlet positioning and pressure. Additionally, chemical modification for cell-adhesive peptides is unsuitable for CM due to the reaction reagents. The RGD peptide itself is edible but too expensive to be a long-term solution. Future work should explore different alginate manufacturing lots, blends, and insoluble additions to further tune mechanical properties, provide dietary or culture supplements, reduce or eliminate RGD modification, and demonstrate success with CM-relevant cell types (e.g., muscle satellite cells, myofibroblasts, adipocytes) from species consumed by humans (e.g., bovine, porcine, avian).

PASTA extrusion is a platform for structured CM. By combining PASTA extrusion with our spinning collector, we produced aligned bundles and demonstrated that PASTA noodles support C2C12 proliferation and differentiation. Culturing myoblasts on these bundles generated aligned, cell-laden structures, a necessary precursor to cultivated steak or other whole cut products. CM research must prioritize scalable production. To that end, we used food-grade alginate: inexpensive, readily available, and already incorporated in food products. Conventional animal meat has zero dietary fiber, and alginate is entirely dietary fiber. Coupled with alginate oligomer benefits, this has the potential to bolster CM’s nutrition and public health advantages. The PASTA noodle bundles themselves are easy to handle due to their aligned, intertwined nature. Ultimately, a culture unit of PASTA noodles about 10–30 cm in diameter would be suitable for structured CM. These findings pave the way for structured CM and warrant further investigation to realize the benefits of this flexible production platform.

## Methods

### Food-grade alginate

Sodium alginate, FCC (CAS 9005-38-3, Lot 2MF0330) was purchased from Spectrum Chemical Mfg. Corp. (New Brunswick, NJ, USA). The polymer measured 97.45% assay with 0.2 mg/kg arsenic, 0.0 mg/kg lead, and 7.59% loss on drying as reported from the manufacturer. While there was no reported data on molecular weight data or M/G ratio from the manufacturer, we performed additional characterization described herein.

### Hydrothermal degradation treatment

Alginate solutions were prepared at 1% w/v in DI water and placed in a convection oven (Forced Air Oven Model BGP-7016 52411-09, Cole-Parmer, Vernon Hills, IL, USA). Solutions were degraded at 70, 80, or 90 °C for 0–72 h. The pH of the hydrothermally degraded alginate solutions was adjusted to approximately neutral with NaOH.

### Sodium alginate gel permeation chromatography

Gel permeation chromatography (GPC) was performed with an Agilent 1260 Infinity HPLC instrument (Agilent Technologies, Santa Clara, CA, USA) with three separation columns (Agilent PLaquagel-OH 30 Å, 50 Å, and 60 Å). The sodium alginate samples were diluted to a target concentration of 2 mg/mL in an aqueous solution of 0.2 M NaNO_3_ + 0.01 M NaH_2_PO_4_ and filtered with nylon syringe filters. The sample molecular weights were quantified against pullulan standards. Each sample was prepared in duplicate and injected twice. Average results are reported.

### Sodium alginate ^1^H NMR

Proton NMR data was collected with the Bruker 800 MHz AVANCE III spectrometer equipped with a CPTCI type cryoprobe. The pulse sequence noesypr1d was used to suppress the water signal. A total of 32–128 scans were accumulated for signal averaging. ASTM International F2259-10^36^ was used to quantify the relative amounts of guluronate (G) and mannuronate (M) monomers.

### Viscosity measurements

The solution viscosity of alginate was measured using 1% w/v solutions at 25 °C using a Discovery HR-2 (TA Instruments, New Castle, DE, USA) and a 1° 20 mm cone geometry at shear rates ranging from 1e3 to 1e4 per second. The resulting data were fit using a Power Law stress model between 1e3 and 1e4 per second shear rate. Samples were measured in triplicate and their means reported.

### Zeta potential

Zeta potential of alginate solutions was determined using a Zetasizer (Nano ZS90, Malvern Instruments, Worcestershire, UK) with the Smoluchowski approximation at 25 °C. Solutions of hydrothermally degraded alginate were diluted to 0.2% w/v then filtered through a 10 µm nylon membrane before loading into a DTS1070 folded capillary zeta cell (Malvern Instruments). The dispersant (water) refractive index was 1.33.

### Alginate preparation for adherent cell culture

To enable cell adhesion, RGD functionalization of the alginate was performed using previously reported carbodiimide chemistry^31,37^. Briefly, alginate was dissolved at 1% w/v in 0.1 M MES buffer with 0.3 M NaCl at pH 6.5. RGD peptide (GGGGRGDSP, Lot U562CCCDG0-1/PE3344; GenScript, Piscataway, NJ, USA) was coupled to carboxyl groups activated with N-hydroxysulfosuccinimide (sulfo-NHS) and 1-ethyl-3-(3-dimethylaminopropyl)carbodiimide (EDC) at a theoretical saturation of 1 in every 50 carboxyl groups (i.e., 2%) of alginate monomers. The reaction was quenched with hydroxylamine hydrochloride, and the solutions were dialyzed using round dialysis tubing with a molecular weight cutoff of 3.5 kDa (Spectra/Por^®^3, Spectrum Laboratories, Inc., Rancho Dominguez, CA, USA). Finally, the dialyzed solutions were sterile filtered and lyophilized until completely dry.

### Degraded alginate storage and loss modulus

Using food-grade sodium alginate degraded at 90 °C for 24 h, we prepared cylindrical discs (8 mm diameter, 1.5 mm height) with a silicone mold as previously described^38^. The discs were prepared with 1% w/v degraded alginate and 500 mM CaCl_2_ solution as a crosslinker. Discs were stored in crosslinker solution for at least 12 h before testing. The storage and loss modulus of alginate gels was measured using a Discovery HR-2 (TA Instruments) and an 8 mm crosshatch geometry. The temperature was controlled at 25 °C. The axial force at the beginning of the test was approximately 0.01 N. Oscillation strain was applied between 4e−3% and 4% at an angular frequency of 10 rad/s. The storage and loss moduli were calculated from the average value between the oscillation strain range of 0.025% and 0.65%.

### Degraded alginate compressive modulus and stress relaxation time

Alginate gels were maintained in crosslinking solution prior to testing. Compression tests were performed using a single-column compressive testing system (Instron 3345, Norwood, MA, USA) at a strain rate of 4% per s, calculated based on initial gel height. The diameter of each gel was measured prior to compression to enable normalization of force values to stress. The compressive modulus was determined from the slope of the linear regression fit between 8-12% strain on the stress-strain curve. For stress-relaxation tests, gels were compressed to 15% strain, and the deformation was held for 60 s with changes in load recorded over this period. Stress relaxation time (*τ*_1/2_) was reported as the time required for the peak force to dissipate to 50% of its initial value^39^.

### PASTA scaffold production

Hydrothermally degraded RGD-modified alginate was dissolved in ultrapure water at a concentration of 1% w/v. Crosslinker solution was prepared using calcium chloride dihydrate at a concentration of 500 mM. Crosslinker solution was placed in a beaker on a stir plate set to 1500 rpm with a custom-designed and 3D-printed spinning collector (see Supplementary Data 7). The alginate solution (250 µL) was loaded into a 1 mL syringe fitted with a 27 G blunt-tip needle (200 µm I.D.) and extruded into the spinning bath. The bundled extrudate (PASTA ‘noodles’) was a solid torus of approximately 1 cm diameter and a circle diameter (thickness) of approximately 5 mm. The PASTA noodle bundles were stored in sterile crosslinker solution until further use.

### PASTA mechanical testing

The tensile strength of individual PASTA noodles was measured using a MicroTester G2 (CellScale Biomaterials Testing, Waterloo, ON, Canada). The instrument was fitted with a custom 3D-printed testing jig and a 6 mm gap (see Supplementary Data 8). The jig was designed using Blender Version 4.2.2 (Blender.org), and the jigs were printed using a Bambu A1 mini (Bambu Labs, Shenzhen, China). PASTA noodles were produced with a 25 G (250 µm I.D.) or 27 G (200 µm I.D.) needle using the spinning collector at conditions stated above. The noodles were separated in water and cut into individual strands for testing. The individual PASTA noodles were secured across the testing gap using paraffin wax. The secured noodles were stored in crosslinker solution before testing, which was performed in ambient air. A 0.4064 mm circular beam was fitted to the MicroTester G2 and the beam traveled 2 mm during the 15 s test duration (0.13 mm/s). The instantaneous stress and strain were calculated using Supplementary Data 5. A strain range of 5 to 10% was used to measure the slope, resulting in the elastic moduli reported. Only linear fits with an *R*^2^ of greater than 0.8 were included in the analysis.

### PASTA diameter measurement

PASTA noodle images were captured using a brightfield microscope (Echo Revolve RVL2-K2, BICO, Göteborg, Sweden) at 1.25× magnification. The images were processed manually using Fiji (ImageJ) software by identifying 10 representative noodle sections approximately 1 mm long and measuring the diameter four times evenly across that length.

### C2C12 cell culture

The murine myoblast cell line C2C12 was expanded in Dulbecco’s Modified Eagle Medium (DMEM, Gibco, USA) supplemented with 5 mM CaCl_2_, 10% v/v fetal bovine serum (FBS, Sigma-Aldrich, St. Louis, MO, USA), and 1% v/v penicillin/streptomycin (P/S, Cat 400-109, lot F323010 GeminiBio, West Sacramento, CA, USA). C2C12 differentiation medium was composed of DMEM supplemented with 5 mM CaCl_2_, 2% v/v heat-inactivated horse serum (New Zealand origin, Gibco, USA), and 1% v/v P/S. The experimental seeding density for proliferation was 7500 cells/cm^2^, and 15,000 cells/cm^2^ for differentiation. The available culture area was calculated assuming the entire extruded volume had the diameter measured as previously described. Seeding was performed with a concentrated cell suspension and left in static culture for 1 h before adding additional media and starting dynamic culture on an orbital shaker (see Supplementary Data 6).

### Staining and immunohistochemistry

Live/dead staining was performed using Calcein AM (Invitrogen, Carlsbad, CA, USA) and propidium iodide (Invitrogen) per manufacturer instructions. Experimental samples were fixed in 4% paraformaldehyde solution supplemented with 50 mM barium chloride (Sigma-Aldrich). Samples were permeabilized with 0.5% v/v Triton X-100, then stained with DAPI (Invitrogen, 1:250) and Alexa-488 Phalloidin (Invitrogen, 1:400) for 45 min. For IHC, a 2 µg/mL solution of anti-fast MyHC (Abcam; Cat #: ab91506, rabbit, 1:500) antibody was prepared in blocking buffer and incubated at 4 °C overnight. Far-red anti-rabbit secondary antibody (647 nm, 1:500 dilution, Abcam; Cat #: ab150083) was incubated at room temperature for 1 h. DAPI and Alexa-488 Phalloidin were added to the staining solution for 10 min. Confocal images were captured using the Leica Stellaris 5 (Leica Microsystems, Wetzlar, Germany).

### Measurement of myogenic gene expression

Collected samples were immediately placed in TRIzol reagent (Invitrogen) and frozen at −80 °C until further processing. Defrosted samples were prepared per manufacturer instructions. The RNA was precipitated and purified with IPA/EtOH/H_2_O and then re-dissolved in RNase-free water (Qiagen, Hilden, Germany). The concentration of RNA was measured with a NanoDrop (Thermo Fisher Scientific, Waltham, MA, USA) and diluted to 15 ng/µL. The diluted RNA was reverse transcribed using Quantiscript Reverse Transcriptase, Quantiscript RT Buffer, and RT Primer Mix (Qiagen) using the manufacturer’s instructions.

The TaqMan PCR reaction was performed in a final volume of 10 µL using Quantitect Probe PCR mix (Qiagen), 37.5 ng cDNA, and a TaqMan probe with FAM reporter dye. The amplifications were performed on an Applied Biosystems™ QuantStudio™ 6 Pro Real-Time PCR System, 96-well, 0.1 mL block (Thermo Fisher). Murine-specific TaqMan Assays *Gapdh* (Mm99999915_g1), *Myod1* (Mm00440387_m1), *Myog* (Mm00446194_m1), and *Myh2* (Mm01332564_m1) were purchased from Thermo Fisher. Results were normalized to the corresponding sample’s endogenous control, *Gapdh*. Reactions were replicated a total of four times and wells that did not amplify were not plotted. Statistical analysis was performed on ∆Ct values. The data distributions were evaluated for normality with the Shapiro-Wilk test. Due to some groups failing the test for normality, a non-parametric Kruskal-Wallis test was used to determine the statistical difference between groups and time points using a *p* < 0.05 criterion.

### Statistical analysis

Statistical analysis was performed using GraphPad Prism version 10.5.0 for Mac (GraphPad Software, Boston, MA, USA). Figures represent mean ± SD. Statistical significance determined with one-way ANOVA assuming a normal data distribution, alpha = 0.05, and Tukey’s correction for multiple comparisons. Significance (*p* < 0.05) is indicated by groups with different letters, while groups sharing the same letters have no significant differences. Analysis details that differ are listed in the associated figure legend.

## Supplementary information

Supplementary Information

## Data Availability

Data sets generated during the current study are available from the corresponding author on reasonable request.

## Code availability

All original code used for data cleaning and analysis is available at https://codeberg.org/alexkermani/PASTA-flow-model-tensile-test and archived at https://doi.org/10.5281/zenodo.20057655.
