# Supplementary material for: Rapid Extrusion of Myotube-Scale Alginate Fibers for Structured Cultivated Meat
Source: NPJ Sci Food. 2026 Jul 21;10:361. doi: 10.1038/s41538-026-00994-2 (PMC13754905; doi:10.1038/s41538-026-00994-2)
Supplement: Supplementary file 1 [file 41538_2026_994_MOESM1_ESM.pdf]

Supplemental information for

Rapid Extrusion of Myotube-Scale Alginate Fibers for Structured Cultivated Meat

Alex S. Kermani[1,2,3,5], Andrea C. Filler[1,4], Edrin C. Miller[1], David E. Block[2,3], J. Kent Leach[1,4]*

[1]Department of Orthopaedic Surgery, UC Davis Health, Sacramento, CA USA 95817
[2]Department of Viticulture and Enology, UC Davis, Davis, CA USA 95616
[3]Department of Chemical Engineering, UC Davis, Davis, CA USA 95616
[4]Department of Biomedical Engineering, UC Davis, Davis, CA USA 95616
[5]Department of Materials Science and Engineering, UC Davis, Davis, CA USA 95616

Supplementary Data 1. Power Law Consistency (K) of alginate solutions (1% w/v) degraded at 70, 80, and 90°C for 72 hours, n=3

	70 °C		80 °C		90 °C	
Deg Time (hours)	**Mean (Pa s^n)**	**SD**	**Mean (Pa s^n)**	**SD**	**Mean (Pa s^n)**	**SD**
0	9.426	0.31624	9.426	0.31624	9.426	0.31624
3	8.628	0.14965	7.503	0.13056	4.654	0.01975
6	8.319	0.07633	6.390	0.09187	2.275	0.02859
12	8.055	0.70831	4.003	0.10875	0.465	0.02095
24	4.689	0.16022	1.704	0.20107	0.050	0.00068
36	4.748	0.23354	0.826	0.02261	0.012	0.00029
48	3.932	0.01721	0.261	0.01522	0.004	0.00003
72	2.354	0.03094	0.097	0.00128	0.001	0.00004

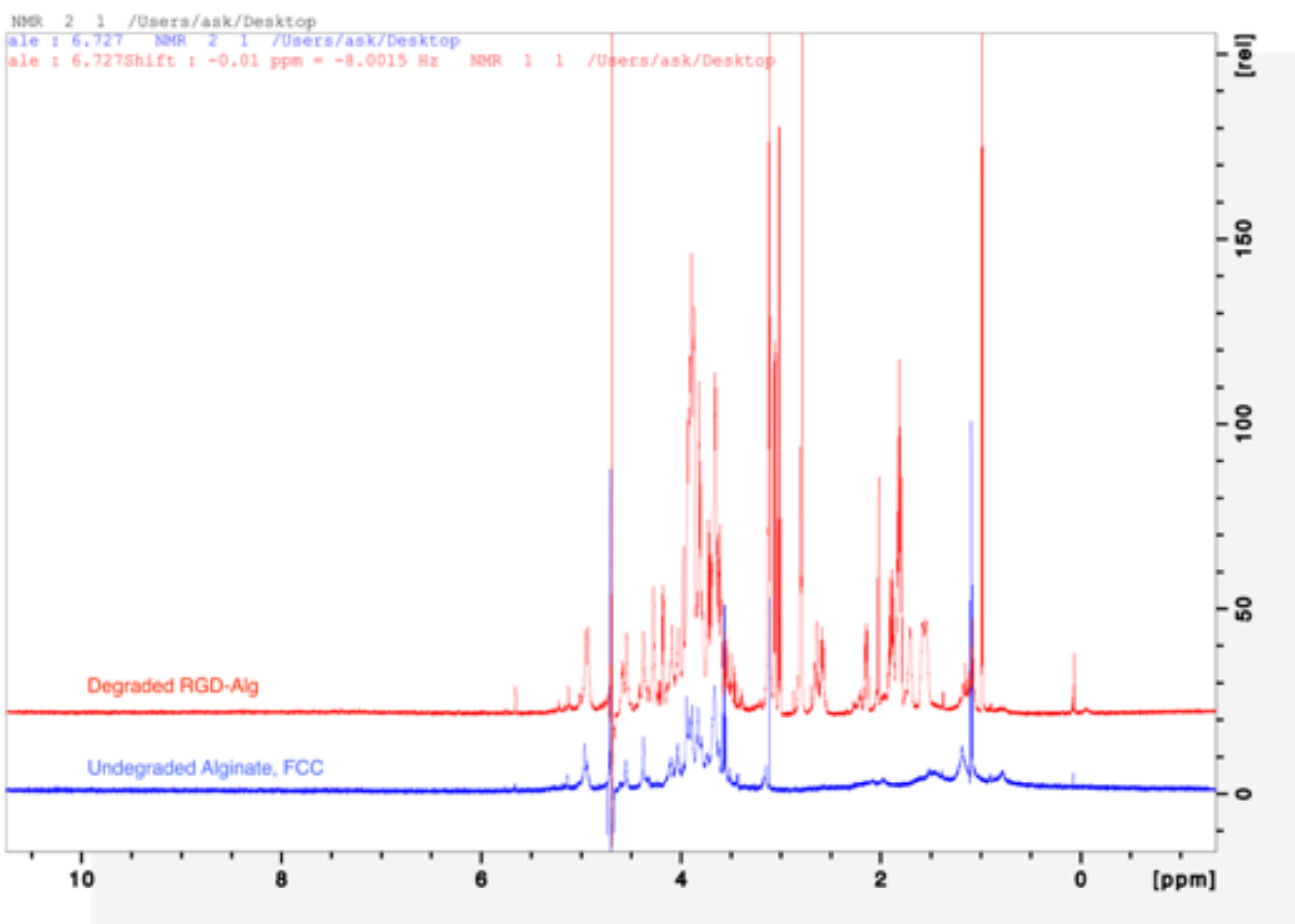

Supplementary Data 2. H1 NMR of degraded, RGD-modified alginate (red) and non-degraded FCC alginate (blue).

Supplementary Data 3. Proton NMR quantification of M and G fractions in undegraded and degraded alginate

	Undegraded, Unmodified	90°C 24 h Degraded, RGD-Modified
**F_G**	0.43	0.51
**F_M**	0.57	0.49
**F_{GG}**	0.21	0.27
**F_{MM}**	0.35	0.26
**F_{MG}**	0.22	0.24
**F_{GGG}**	0.06	0.17
**F_{MMM}**	0.07	0.13
**F_{GGM}**	0.15	0.11

Supplementary Data 4. PASTA inner diameter and resulting noodle diameter (n=24) using 1% w/v degraded alginate (90°C 24 h)

			Noodle Diameter	
**Tip Color**	**Tip Gauge**	**Inner Diameter (µm)**	**Mean (µm)**	**SD**
Yellow	32	100	90.5	10.8
Lavender	30	150	102.7	12.0
White/Clear	27	200	115.8	26.9
Red	25	250	176.7	32.8
Orange	23	330	215.6	33.0
Blue	22	410	281.0	47.4
Pink	20	610	477.0	46.0

Supplementary Data 5. Equations used to calculate tensile modulus

LaTeX Equation	Equation	Description
`\text{Strain }(\epsilon) = \frac{L - W/2}{W}`	$\text{Strain }(\epsilon) = \frac{L - W/2}{W}$	Tensile stress
`\text{Stress }(\sigma) = \frac{F_{\text{fiber}}}{\text{Area}}`	$\text{Stress }(\sigma) = \frac{F_{\text{fiber}}}{\text{Area}}$	Tensile strain
`F_{\text{fiber}} = \frac{F_{\text{beam}}}{2}\frac{L}{\Delta d}`	$F_{\text{fiber}} = \frac{F_{\text{beam}}}{2}\frac{L}{\Delta d}$	Tensile force resolved along a single side of the fiber
`L = \sqrt{(\Delta d)^2 + (W/2)^2}`	$L = \sqrt{(\Delta d)^2 + (W/2)^2}$	Fiber length calculated with right angle geometry

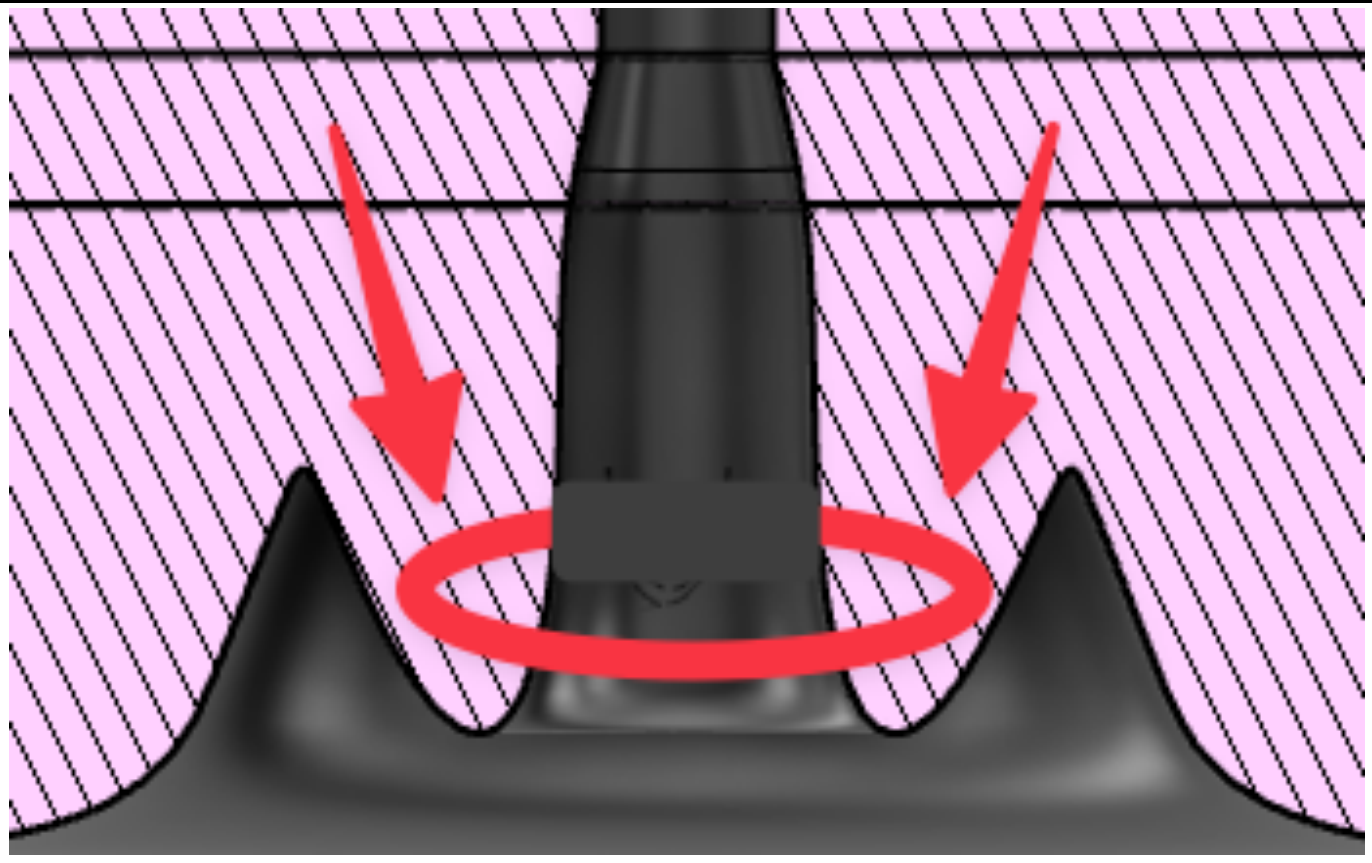

Supplementary Data 6. PASTA cell culture well cross-section. Arrows indicate the trough and the oval indicates the orientation of the PASTA scaffold. The grey color is agarose, and the pink is void volume in the culture well.

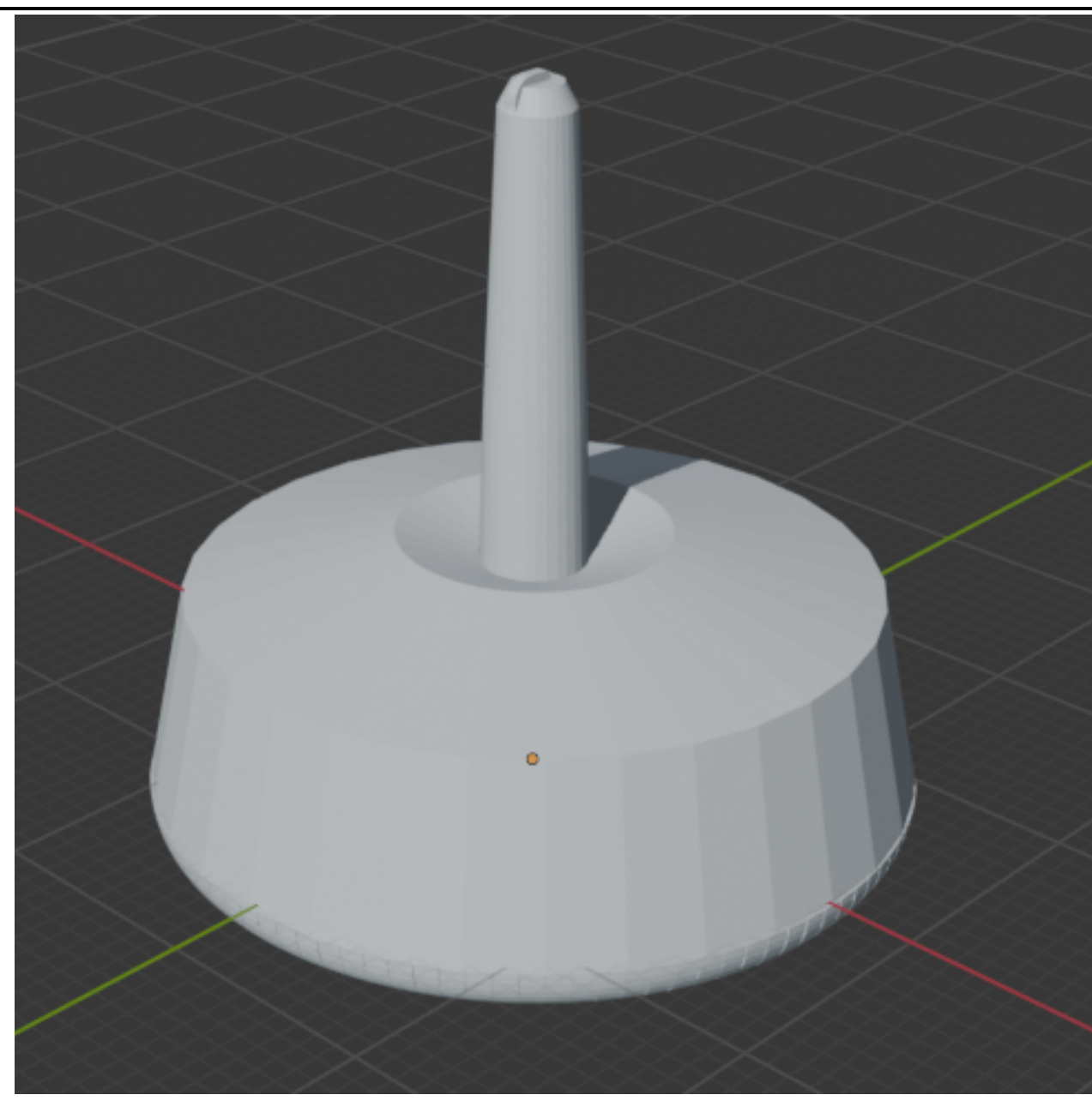

Supplementary Data 7. Rendered image of spinning collector produced with Blender.

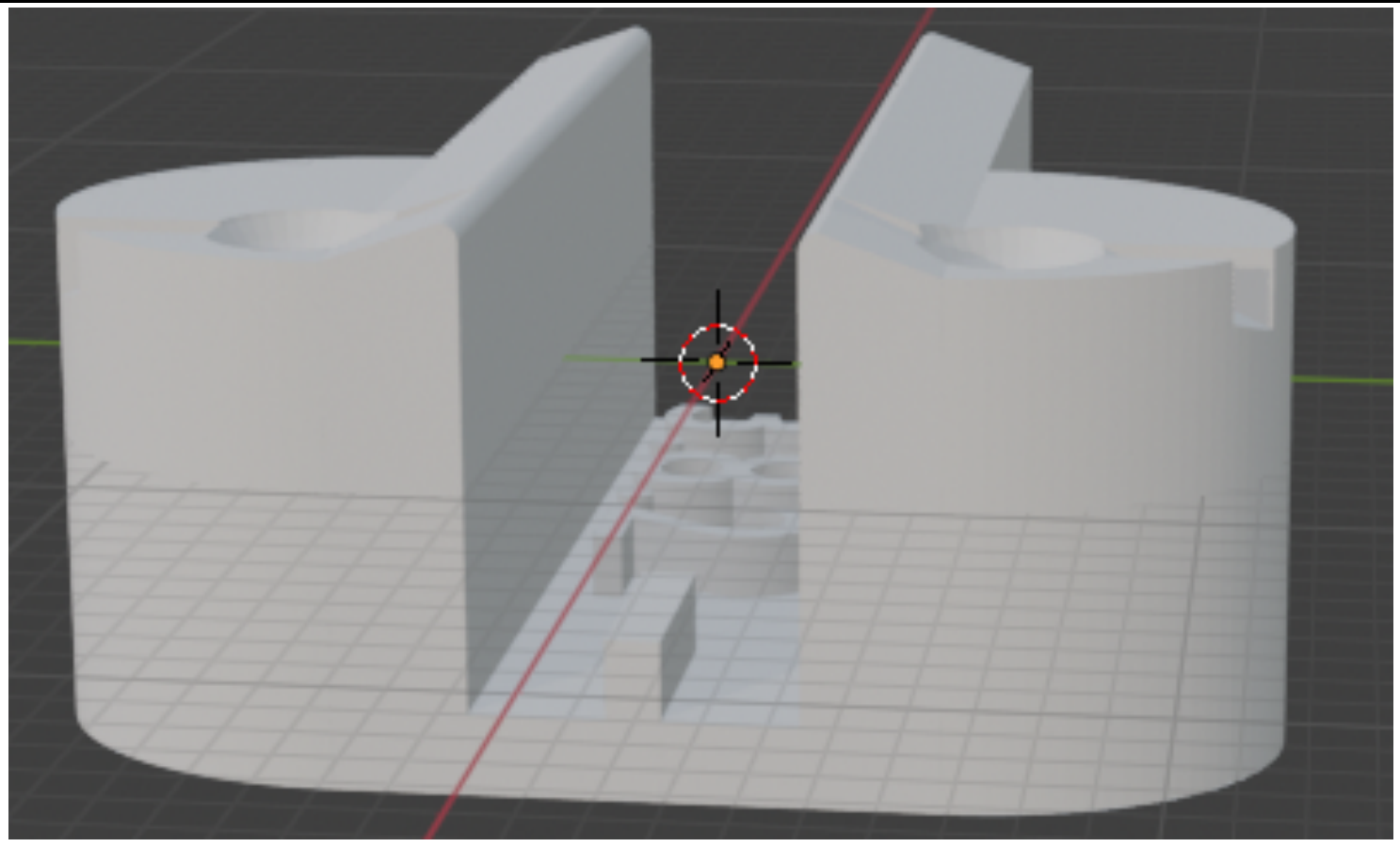

Supplementary Data 8. Rendered image of tensile testing jig produced with Blender. The PASTA noodle is suspended across the near gap and anchored in the divots with wax.

Additional Supplementary Data, including tensile testing processing script with example data, Blender file, and our interactive flow modeling tool can be found at https://codeberg.org/alexkermani/PASTA-flow-model-tensile-test and archived at https://doi.org/10.5281/zenodo.20057655
